# Advancing patient-centered research practices in a pragmatic patient-level randomized clinical trial: A thematic analysis of stakeholder engagement in Emergency Medicine Palliative Care Access (EMPallA)

**DOI:** 10.1186/s40900-023-00539-x

**Published:** 2024-01-23

**Authors:** Nicole Zhao, Allison M. Cuthel, Owen Storms, Raina Zhang, Rebecca Liddicoat Yamarik, Jacob Hill, Regina Kaur, Kaitlyn Van Allen, Mara Flannery, Alex Chang, Frank Chung, Sumeet Randhawa, Isabel Castro Alvarez, Angela Young-Brinn, Constance L. Kizzie-Gillett, Dawn Rosini, Eric D. Isaacs, Ernest Hopkins, Garrett K. Chan, Juanita Booker-Vaughns, Margaret Maguire, Martha Navarro, Neha Reddy Pidatala, Patrick Dunn, Pluscedia Williams, Robert Galvin, Romilla Batra, Sally Welsh, William Vaughan, Jean-Baptiste Bouillon-Minois, Corita R. Grudzen

**Affiliations:** 1 Renaissance School of Medicine at Stony, Brook University, Stony Brook, NY USA; 2 Ronald O. Perelman Department of Emergency Medicine, New York University Grossman School of Medicine, 227 East 30th Street, New York, NY 10016 USA; 3 Department of Medicine, Tibor Rubin Long Beach Veteran Affairs, Long Beach, CA USA; 4 Department of Wellness and Preventive Medicine, Cleveland Clinic, Cleveland, Ohio USA; 5 Charles R. Drew University of Medicine and Science, Los Angeles, CA USA; 6 Lillie’s Circle of Care, Streamwood, IL USA; 7 University of Florida Shands Hospital, Gainesville, FL USA; 8 The University of Florida College of Medicine, Gainesville, FL USA; 9 Department of Emergency Medicine, University of California San Francisco, San Francisco, CA USA; 10 The Phoenix Group Foundation, Atlanta, GA USA; 11 HealthImpact, Oakland, CA USA; 12 Cambia Health Solutions, Portland, OR USA; 13 American Cancer Society, Atlanta, GA USA; 14 American Heart Association, Dallas, TX USA; 15 The Lundquist Institute/Harbor-UCLA Medical Center, Torrence, CA USA; 16 The Blackstone Group, New York, NY USA; 17 Senior Care Action Network (SCAN) Health Plan, Long Beach, CA USA; 18 Hospice and Palliative Nurses Association, Carnegie, PA USA; 19 Patient Advocacy, Fairfax, VA USA; 20 Emergency Department, University Hospital of Clermont-Ferrand, 63000 Clermont-Ferrand, France; 21 Division of Supportive and Acute Care Services, Memorial Sloan Kettering Cancer Center, New York, NY USA

**Keywords:** Study Advisory Committee, Palliative care, Stakeholder participation, Research design, Patient and public involvement

## Abstract

**Background:**

Involving patient and community stakeholders in clinical trials adds value by ensuring research prioritizes patient goals both in conduct of the study and application of the research. The use of stakeholder committees and their impact on the conduct of a multicenter clinical trial have been underreported clinically and academically. The aim of this study is to describe how Study Advisory Committee (SAC) recommendations were implemented throughout the Emergency Medicine Palliative Care Access (EMPallA) trial. EMPallA is a multi-center, pragmatic two-arm randomized controlled trial (RCT) comparing the effectiveness of nurse-led telephonic case management and specialty, outpatient palliative care of older adults with advanced illness.

**Methods:**

A SAC consisting of 18 individuals, including patients with palliative care experience, members of healthcare organizations, and payers was convened for the EMPallA trial. The SAC engaged in community-based participatory research and assisted in all aspects from study design to dissemination. The SAC met with the research team quarterly and annually from project inception to dissemination. Using meeting notes and recordings we completed a qualitative thematic analysis using an iterative process to develop themes and subthemes to summarize SAC recommendations throughout the project’s duration.

**Results:**

The SAC convened 16 times between 2017 and 2020. Over the course of the project, the SAC provided 41 unique recommendations. Twenty-six of the 41 (63%) recommendations were adapted into formal Institutional Review Board (IRB) study modifications. Recommendations were coded into four major themes: Scientific, Pragmatic, Resource and Dissemination. A majority of the recommendations were related to either the Scientific (46%) or Pragmatic (29%) themes. Recommendations were not mutually exclusive across three study phases: Preparatory, execution and translational. A vast majority (94%) of the recommendations made were related to the execution phase. Major IRB study modifications were made based on their recommendations including data collection of novel dependent variables and expanding recruitment to Spanish-speaking patients.

**Conclusions:**

Our study provides an example of successful integration of a SAC in the conduct of a pragmatic, multi-center RCT. Future trials should engage with SACs in all study phases to ensure trials are relevant, inclusive, patient-focused, and attentive to gaps between health care and patient and family needs.

*Trial Registration*: Clinicaltrials.gov Identifier: NCT03325985, 10/30/2017.

## Abstract (plain language summary)

Clinical research should involve patient and community stakeholder perspectives to make sure the study addresses questions important to the studied population. One way to do this is by creating a group of stakeholders who can advise on the conduct of a study. We assembled a Study Advisory Committee (SAC) for the Emergency Medicine Palliative Care Access (EMPallA) trial. The purpose of this clinical trial is to compare the effectiveness of nurse-led telephonic case management and specialty, outpatient palliative care of older adults with advanced illness. This paper describes how the SACs involvement translated into direct impacts on the EMPallA trial. The trial research team held regular meetings with the SAC throughout the trial process. Their involvement led to many significant changes in the trial, such as  expanding recruitment inclusion criteria (Spanish-speaking patients), and including survey instruments to measure lonelines and caregiver burden. The SAC also devised strategies to overcome patient and caregiver recruitment and retention challenges, including the creation of patient-friendly materials and training for research coordinators. This study provides a successful example of how actively engaging patient and community stakeholders, through committee engagement, can promote patient priorities in all phases of a trial while facilitating patient recruitment and retention.

## Background

Actively engaging patient and community stakeholders in the design of clinical trials is crucial to addressing questions and bridging gaps in patient-centered research that are relevant to both the investigator and target populations [1, 2]. Specifically, patients and stakeholders can steer the design of clinical trials to be effective in disseminating and implementing findings into standard practice [1]. This adds to overall patient satisfaction and uses genuine feedback to continuously improve patient outcomes [3, 4]. The patient and community perspective prioritizes patient goals, which may not always be apparent to researchers, and ensures studies hold up to their ethical standards [4]. Furthermore, there is a moral imperative to include patient and community perspectives in research to ensure patients are protected during research. Healthcare organizations that represent and advocate for illness groups such as heart failure give valuable insight into how these patients will be best served by the study. Payor involvement can give insights into study design and dissemination that can improve payor uptake of results upon study completion. As healthcare models further aim to implement patient-centered care, examination of the impact of patient and community perspectives on research design and implementation is crucial.

The Patient-Centered Outcomes Research Institute (PCORI) has transformed clinical research with its unique commitment to funding comparative clinical effectiveness research and mandating a plan for stakeholder engagement [5]. PCORI defines patient and stakeholder engagement as the meaningful involvement of patients and stakeholders in developing the research question, relevant outcomes to be studied, participant characteristics, protocols, data collection, interpretation of results, and dissemination of conclusions [6]. While many studies understand the importance of incorporating the patient perspective, the use of patient and community stakeholder committees and their impact on the conduct of a multicentered clinical trial have been understudied and underreported [7].

The Emergency Medicine Palliative Care Access (EMPallA) trial is a randomized, pragmatic clinical trial comparing the effectiveness of specialty, outpatient to nurse-led telephonic palliative care for older adults with advanced illness initiated in the emergency department (ED). The EMPallA trial recruited 1,350 patients across eighteen EDs (nine states across the United States (US)) and their respective caregivers. The parent EMPallA study evaluated the effectiveness of each intervention by comparing patient quality of life, healthcare utilization, loneliness, symptom burden, as well as caregiver strain, quality of life, and bereavement [8].

A Study Advisory Committee (SAC) was assembled at the project’s inception and consisted of a variety of patient and community stakeholders (patients with palliative care experience, members of healthcare organizations, and payers). The goal of the SAC was to assist in all stages of the research, including the trial design and planning, recruitment, implementation, and dissemination of study conclusions [8]. Using a thematic analysis approach, we describe recommendations the SAC provided that were implemented over the course of EMPallA.

## Methods

This study was approved by the New York University Grossman School of Medicine Institutional Review Board (ID# s17-01211 and s19-00419).

### Study design

We used a descriptive case study research design to describe SAC recommendations that have been implemented throughout the EMPallA trial. We completed a qualitative thematic analysis using an iterative process to develop themes and subthemes to summarize the data. After themes were identified, we used a pre-existing framework, Shippee et al. to classify our findings into three study phases (preparatory, execution, translational) [9]. The goal of using the Shippee et al. framework was to better understand when in the research project most types of recommendations occurred (ex. Did all study design related recommendations occur in year 1 and/or preparatory phase?). We engaged in an iterative process to generate patterns in the data until data was deemed saturated. Our four major themes demonstrate how SAC recommendations translated to impactful study modifications.

### Study participants and recruitment

A purposive sampling method was used. Initial stakeholders from all three stakeholder categories were recruited to participate on the EMPallA SAC based on (1) their history of commitment to patient-centered outcomes research (2) previous collaboration with the EMPallA Principal Investigator (PI) (co-author CRG) and (3) in accordance with the need for demographically and geographically diverse representation. Further stakeholders were identified by word-of-mouth recommendation.

EMPallA’s SAC consisted of 18 members from across the US (n = 7 Eastern US, n = 8 Pacific US, n = 3 Central US) representing three major stakeholder categories: patients (n = 3) experiencing serious diseases or their caregivers (n = 4), members of healthcare organizations involved with study-related illnesses and/or palliative care (n = 5), and payers (n = 6). Examples were officers of associations such as the American Heart Association and the American Cancer Society, the chief medical officer of a Medicare Managed Care plan, officers of healthcare foundations, and community faculty of a historically black university. The SAC was composed of 12 women and six men representing Black, Asian, and Latino communities (50% White non-Hispanic, 28% Black non-Hispanic, 17% Asian, 6% White Hispanic).

### Study procedures

Data were collected between December 2017 and November 2020 and included SAC meeting notes from quarterly, annual, and ad-hoc meetings along with supplemental audio and video recordings. Since the EMPallA project’s inception (10/30/2017), the SAC met both quarterly and annually, with all meetings facilitated and led by the New York University (NYU) research team (CRG, MD, female, clinician and researcher). The quarterly meetings occurred three times per year via conference call (either Webex or Zoom) for approximately 60–90 min. Annual meetings were longer and ranged between 4 and 8 h. Annual meetings originally occurred in-person (2017–2019) and transitioned to virtual thereafter due to the COVID-19 pandemic. All meetings followed a general structure: presentations and updates by research team members; discussion of current barriers  and facilitators; open forum discussion. Ad hoc meetings also occurred and ranged from 30 to 60 min.

The research team was responsible for writing and disseminating meeting notes to the SAC within 48 h after each SAC meeting to facilitate transparency and to allow member checking for additional reflection and feedback. We did not record which individual in the SAC voiced a specific recommendation, but all final recommendations were made by consensus of the full SAC group. Given the pre-established level of trust and rapport leveraged from previous work experiences, patients and caregivers were very engaged during the meetings, often directing the discussion more than the payers and healthcare organization participants. Members of the SAC were contracted as paid consultants which allowed them to receive stipends for their support and travel reimbursement, when applicable.

### Analysis

All sixteen available SAC meeting notes, audio, and video recordings were transcribed. The coders, two EMPallA Project RCs (co-authors SR, IC) received the full dataset in 2021 and coding and data analysis took place between August 2021 and July 2022. The Senior Research Project Manager (SRPM)(AMC), a trained qualitative researcher, oversaw the qualitative research process. Both RCs (SR, IC) independently reviewed all sources of data, then coded the content highlighting potentially relevant phrases and sentences related to the research question (study modifications and recommendations), and initial codes were generated. The development of the codebook was an iterative process and the team (AMC, SR, IC) came together to reach a consensus for coding interpretation. If necessary, the SRPM would make the final decision if consensus on coding was not reached. Deductive themes and subthemes were generated. Using the Shippee et al. framework the team indexed the coded recommendation into relevant study phases [9]. Codes were condensed into meaningful themes. Upon completion of the thematic analysis and classification into relevant Shippee study phase, the coding team next completed a crosswalk exercise to match which specific approved Institutional Review Board (IRB) modifications were a direct result from a coded SAC recommendation. Results were compiled and organized into a single table (Table 1). Participant checking was accomplished via e-mail with all members of the SAC.

**Table 1:** Crosswalk of Study Advisory Committee (SAC) recommendations, Coded Themes/Subthemes, Shippee framework phase and Institutional Review Board (IRB) modification

Recommendation number	SAC recommendations and suggestions	Theme	Subtheme	Shippee framework phase*	Modification made based on SAC recommendation	Required IRB modification
1	Investigate how caregivers cope	Scientific	Design advice	I.1	Implemented the short-form Zarit Caregiver Burden survey into screening tool at 3, 6, and 12-month follow-up surveys	Yes
2	Consider the geographic location differences of the enrolled sites. How do you reach patients in rural areas where outpatient palliative care clinics are not available?	Scientific	Conceptual suggestion	I.1	This recommendation was provided prior to the implementation of telehealth outpatient visits as enrolling sites are all primarily located in urban or suburban locations. In the patient enrollment forms the research team captured information related to the patient’s home address and the address of the outpatient clinic and the team is anticipating analyzing if distance to/from the clinic was a barrier. Additionally, the pandemic provided an opportunity to revisit care delivery techniques and allowed patients to receive outpatient palliative care via telehealth	Yes
3	Explore the potential barriers to palliative care access that may impact engagement across the intervention arms (e.g. distance from clinic/services, income etc.)	Scientific	Design advice	I.1, II.2, II.3, II.4	This recommendation was incorporated into data collection and analysis plans. For example, survey instruments were updated to ensure potential barriers were collected and analysis plans were updated to account for different barriers	Yes
4	Update materials to include more exciting terms so patients do not associate palliative care with death	Scientific	Recommendation for supplementary study materials	II.1	RCs were trained on the appropriate methods of describing palliative care with patients and caregivers	No
5	Implement a satisfaction survey for patients in an effort to improve the intervention	Scientific	Design advice	II.1	No patient satisfaction survey was formally collected as this was outside the scope of the research study. Nonetheless, any time study participants provided feedback it was brought to the research team’s attention and discussed	No
6	Expand inclusion criteria to Spanish Speaking participants	Scientific	Design advice	II.1	The recruitment criteria was expanded to include Spanish Speaking participants	Yes
7	Consider adjusting protocols for patients with disabilities specifically mental disabilities and other conditions that impact this group of study participants (ex. Dementia)	Scientific	Design advice	II.1	In the screening tool, a dementia question was added to assess whether dementia was included on the patients’ active problem list in Epic, signaling exclusion. RCs made accommodations for any patients who required by prioritizing certain questions within the follow-up surveys, requesting presence of caregiver during survey completion etc	Yes
8	Explore how the outpatient study arm will measure loneliness	Scientific	Design advice	II.1	The team incorporated the validated University of California Los Angeles (UCLA) Three-Item Loneliness Scale into the 3, 6, and 12 month patient follow up surveys to measure loneliness for both study arms	Yes
9	Ensure patients or caregivers are familiar with the study and a telephonic nurse may be calling them (if enrolled in that study arm)	Scientific	Recommendation for supplementary study materials	II.1, II.2	A welcome packet was developed and sent to each patient and caregiver enrolled	Yes
10	Provide each patient with a welcome packet that is bright and at an appropriate reading level	Scientific	Recommendation for supplementary study materials	II.1, II.2	A patient facing brochure was developed in partnership with the SAC members	Yes
11	Enrolled caregivers should feel part of the research process	Scientific	Design advice	II.1, II.2	A separate caregiver screener with questions specific to caregivers’ emotions, feelings, health was implemented	Yes
12	Shorten welcome letters, as drafts were too lengthy	Scientific	Recommendation for supplementary study materials	II.1, II.2	With the SAC’s feedback, the patient facing brochure was updated to include a shortened welcome letter. All patient facing materials were reviewed by the SAC	Yes
13	Have patient-facing visuals for palliative care at the time of recruitment to provide patients and caregivers with an overview of the study	Scientific	Recommendation for supplementary study materials	II.1, II.2	In partnership with the SAC members, the research team created a visual describing palliative care that is reader friendly	Yes
14	Include more images than text in patient facing materials	Scientific	Recommendation for supplementary study materials	II.1, II.2	RCs continued to assist patients where needed, and incorporated visuals of the study timeline alongside EMPallA descriptors that were required to be provided by regulatory guidelines	No
15	Replace the use of the word "caregiver" with other similar words that are more sensitive to the population	Scientific	Recommendation for supplementary study materials	II.1, II.2	Theae changes were implemented throughout study materials and communicated to research coordinators. Instead of “caregiver, the terms supportive care, companion, helper, support system were used	Yes
16	Use the terminology “stress” instead of “burden” when communicating with patients and caregivers	Scientific	Recommendation for language use	II.1, II.2	Language was adapted in the REDCap screener, scripts, and subsequent follow-up surveys	Yes
17	Implement telehealth video option during pandemic when clinics were significantly limiting in-person visits to reduce risk of COVID-19 for this vulnerable population	Scientific	Design advice	II.1, II.2	This recommendation resulted in a major protocol change. Originally, outpatient clinics only met patients randomized in this study arm in-person, but the study was adapted to allow telehealth visits	Yes
18	Create a video to introduce the study to the patient and caregiver to enhance recruitment	Scientific	Recommendation for supplementary study materials	II.2	Due to limited resources a video could not be developed, but the research team created resources summarizing the study for all patients and caregivers, including visual timelines and full descriptions	No
19	Shorten recruitment scripts	Scientific	Recommendation for supplementary study materials	II.2	As scripts were developed with IRB regulatory guidelines in mind and could not be modified as consistent recruitment processes had to be standardized across all sites	No
20	Be mindful of resources available in each enrolling Emergency Department	Pragmatic	Implementation advice	I.1	Research teams were encouraged to train RCs on respective sites’ available resources. RCs were also encouraged to share unique resources during peer-to-peer meetings that may be helpful to their partnering sites	No
21	Understand the impact of spiritual differences	Pragmatic	Implementation advice	I.1 II.1	A standard religion question was added in the screening survey. The specific question added was “Are you a member of a faith community?”	Yes
22	Be conscientious of where the patient is from and their unique healthcare needs	Pragmatic	Implementation advice	I.1, II.1	Each RC was provided open user rights within the electronic health record to better understand each patient enrolled (example, a RC was able to see if the patient was currently in the hospital, or if they had a recent encounter with a physician etc.)	Yes
23	Develop a phone script that adequately addresses caregiver duress	Pragmatic	Recommendation for language use	II.1	Research Coordinators (RCs) were trained by SAC members on appropriate language to use while speaking with caregivers	No
24	Implement pre-visit reminder calls	Pragmatic	Implementation advice	II.1	Before each Outpatient visit, the RCs would remind patients of their upcoming appointment. This data was documented in the REDCap Outpatient Log	Yes
25	Do not e-mail gift cards to participants	Pragmatic	Advice about dissemination	II.1	Given the unique patient population recruited for this study, the research team worked with their internal finance team to provide incentive gift-cards either in-person or physically sent in the mail	Yes
26	Provide more resources directly to caregivers to help them understand the benefits of palliative care	Pragmatic	Implementation advice	II.1	SAC members who served as caregiver advocates trained the RCs the most appropriate ways to interact with caregivers	No
27	Integrate the use of a translator services as they are used during emergency department visits, and Spanish-speaking patients should be able to use this mode of communication	Pragmatic	Implementation advice	II.1, II.2	The research coordinator protocol was updated with information related to using the translator phone	Yes
28	When recruiting in-person (with patient’s verbal permission), use their phone to contact their caregiver to see if they are interested in participating in the study. This was to avoid barriers related to robo and spam calls, and the caregivers not picking up their phone from an unknown telephone number	Pragmatic	Implementation advice	II.1, II.2	This suggestion was adapted and research coordinators across all recruitment sites when appropriate would use the patient’s telephone to directly call their caregiver to see if they were interested in participating in the study	Yes
29	Follow-up with each participant after study enrollment to keep patient engaged for the duration of the 6-month intervention and 12-month data collection	Pragmatic	Implementation advice	II.1, II.2, II.3	A postcard was created and was mailed and or provided to each participant after enrollment reminding them about the study benefits and expectations. Research coordinators would also call participants	Yes
30	Explain to the patient the definition of a caregiver before asking patient if s/he has a caregiver who wants to participate in the study	Pragmatic	Recommendation for language use	II.1, II.3	Recruitment scripts were updated, and discussions related to overcoming hurdles in recruiting caregivers were covered in biweekly peer-to-peer research coordinator learning collaboratives	Yes
31	Have research staff assist in coordinating with patient’s other physicians	Pragmatic	Implementation advice	II.2	During the recruitment process, each RC would enter the contact information for the patient’s providers in the study’s secure database REDCap. If the patient was enrolled in the telephonic study arm the nurse would assist with care coordination processes	Yes
32	Ensure that Outpatient Palliative Care clinics are well staffed	Resource	Staffing infrastructure suggestion	I.1	No changes could be made to the staffing infrastructures at each external outpatient clinic, but the research team worked with outpatient palliative care clinics to understand staffing trends, hiring updates and strategic interim plans to ensure that capacity will allow for EMPallA patient care	No
33	Hire additional Spanish speaking research coordinators at each enrolling site	Resource	Staffing infrastructure suggestion	II.1, II.2	Due to budgetary constraints and inability to find appropriate staff this was not feasible at each enrolling institution. Nonetheless, the research team recommended that bi-lingual qualifications be included in hiring criteria. Where this could not be accommodated, research teams made use of hospital translator phones to ensure that the Spanish-speaking population could accurately be reached	No
34	Ensure all Spanish patient facing materials are culturally sensitive	Resource	Patient-centered suggestion	II.1, II.2	All materials were directly translated from original documents to maintain a standardized tone and reviewed by Spanish speaking Latinx SAC member	Yes
35	Implement mock Spanish recruitment trainings	Resource	Patient-centered suggestion	II.1, II.2	Leveraging our SAC member with expertise in recruiting Spanish-speaking participants, the research team implemented focused trainings on recruiting Spanish-Speaking participants between the SAC member and each individual research coordinator responsible for recruitment	Yes
36	Implement mock recruitment training for new enrollment sites that were added to the study design	Resource	Patient-centered suggestion	II.1, II.2	The research team scheduled time between SAC members and not only new enrollment sites, but also newly hired RCs to conduct mock recruitment	No
37	Involve Patient stakeholders to train new research team members via WebEx conferencing on the research study pitch language used to describe palliative care	Resource	Patient-centered suggestion	II.1, II.2	SAC members trained RCs on a routine basis to ensure that staff on the ground was provided with updated resources throughout study enrollment	No
38	Assess the feasibility of covering patient’s physician costs	Resource	Patient-centered Suggestion	II.2	Patient Centered Outcome Research Institute (PCORI) funded project cannot cover physician related costs, but this conversation was the impetus of adding “insurance status” as a variable in the patient screening process	Yes
39	When reviewing data internally (SAC and research team), update bar graphs to include patient and caregiver enrollment numbers on the same bar graph	Dissemination	Advice about dissemination	II.4	The research team’s Data Analyst updated this figure to consistently report patient and caregiver enrollment targets and goals on the same bar graph for visual ease	No
40	When reviewing data internally (SAC and research team), always include ethnicity enrollment breakdown for patients and caregivers	Dissemination	Advice about dissemination	II.4	The research team’s Data Analyst consistently provides race/ethnicity data when presenting enrollment updates during the internal team meeting	No
41	Disseminate study progress to patients while the study is ongoing. One Suggestion included having a website for patients to see enrollment numbers and target goals, results etc	Dissemination	Advice about dissemination	III.1	Once primary data collection and analysis concludes, study results will be disseminated to study participants	No

*Framework Element represents the phases and stages of the Shippee et al. framework, defined as:

I.1 = Preparatory Phase, Agenda Setting and Funding

II.1 = Execution Phase, Study Design and Procedures

II.2 = Execution Phase, Study Recruitment

II.3 = Execution Phase, Data Collection

II.4 = Execution Phase, Data Analysis

III.1 = Translational Phase, Dissemination

PCORI, Patient-Centered Outcome Research Institute; SAC, Study Advisory Committee; RC, Research Coordinator; EMPallA, Emergency Medicine Palliative Care Access

#### Theoretical framework

**Fig. 1:**
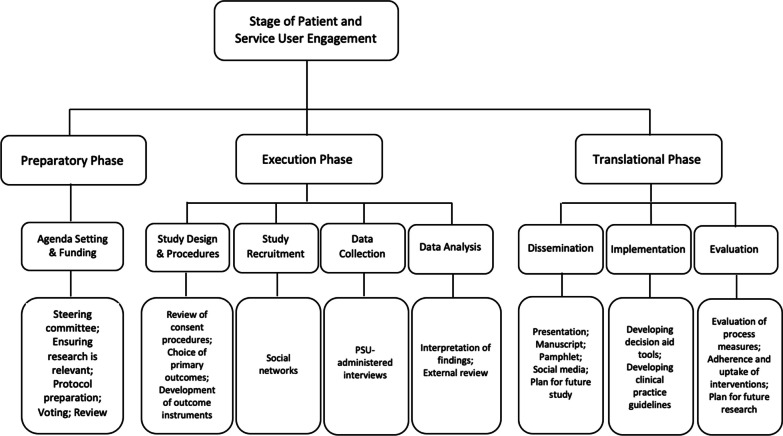
Shippee et al. framework for patient and service user engagement

The Shippee, et al. framework, (Fig. 1) was used to understand longitudinally at what phase within the project the recommendations occurred [9]. Specifically, this framework standardizes the structure of reporting the SAC’s recommendations into three broad research design phases: preparatory, execution, and translational. The preparatory phase focuses on agenda setting by ensuring the study’s novelty and relevance, as well as creating effective protocols to explore research questions. The execution phase focuses on subthemes of study design and procedures, study recruitment, data collection, and analysis. Lastly, the translational phase focuses on dissemination, implementation, and evaluation of the study’s conclusions. Each phase is further categorized into stages to delineate research activities impacted by stakeholder engagement.

## Results

Sixteen SAC meetings occurred between December 29, 2017–November 13, 2020. All meetings were used in the analysis. In half of the meetings (8/16) the SAC members provided discrete recommendations. The remaining meetings were used to discuss implementation of the suggested recommendations, barriers/facilitators, or other project related topics. A total of 41 recommendations, which subsequently resulted in 26 distinct IRB study modifications were made during the specified timeframe. All study protocol modifications were accepted by the funder to ensure the main research question, subsequent aims, and overall study design remained consistent and had the same scientific rigor as proposed in the original grant application. As such, despite changes made to the study protocol, patients recruited at the end of the study were not demonstrably different in terms of age, race/ethnicity, gender, or disease category.

**Fig. 2:**
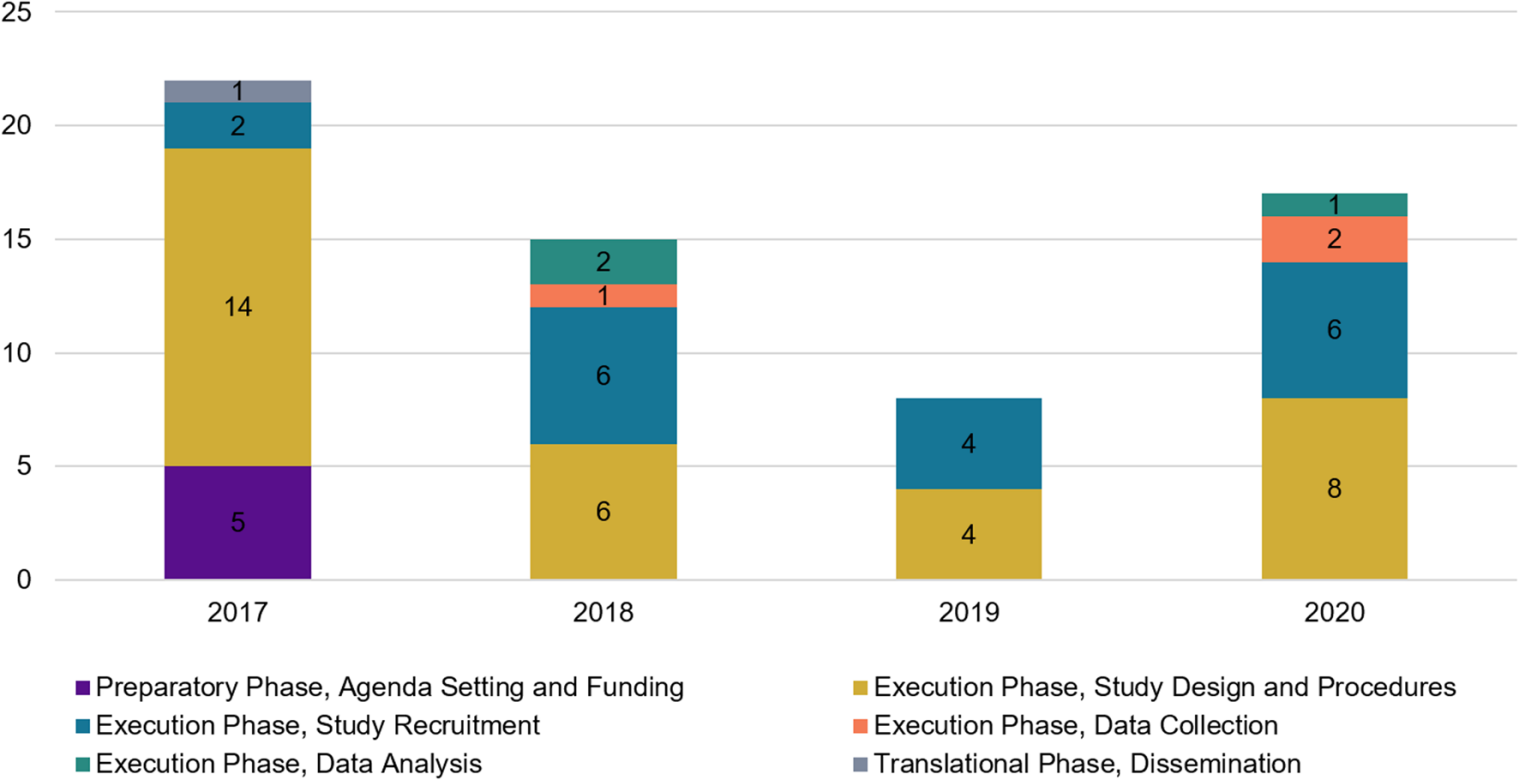
Longitudinal breakdown of the number of SAC recommendations across meetings (by calendar year) and organized by Shippee et al. framework categories. *Data is not mutually exclusive and recommendations were coded in more than one category

Four major themes emerged in the data (Scientific, Pragmatic, Resource and Dissemination) and all coded data were organized using the Shippee, et al. framework. Results reflect both the major themes through the thematic analysis and also the Shippee framework three main study phases: Preparatory, execution, and translation [9]. Data demonstrating the non-linear nature of SAC recommendations (organized by meeting year) and engagement throughout the project can be found depicted in Fig. 2. Data in Fig. 2 were not mutually exclusive to a specific phase or stage and thus, were included in multiple categories. A vast majority (94%) of IRB modifications made due to SAC recommendations were indexed in the execution phase of the framework. Few recommendations were related to the preparatory or translational Phases. The translational phase of the framework includes the stages of dissemination (Fig. 1) [9]. Given the SAC members were funded on the EMPallA project for a specific timeframe (study design through the end of data collection phases), only one recommendation during the data collection period pertained to the translational phase (Recommendation #41, Table 1). This occurred during the first SAC meeting when the SAC suggested study updates and results be disseminated to patients in real-time. No IRB adaptations were made based on this recommendation as the research team could not analyze or disseminate results until recruitment and data collection were complete (July 2023).

Below we describe exemplary SAC recommendations within each coded theme and subthemes and how each recommendation was translated to meaningful changes, or “actions,” in the implementation of the EMPallA trial. The full list of SAC recommendations (including recommendations both made and not made), their associated IRB modifications, Shippee framework phase classifications, and themes and subthemes can be found in Table 1. Within Table 1 we also provide details related to which modifications did not require an IRB modification and/or the rationale on why a recommendation was not implemented.

### Theme 1—Scientific

The Scientific theme was related to changes that would have impacted the original research plan, scientific design, or items related to describing the study to participants. The most common subthemes were design advice and recommendations for supplementary study materials.

#### Subtheme: design advice

##### SAC recommendation

In setting research priorities, the SAC proposed the research team measure how caregivers cope with their caregiver’s (relative, friend, partner etc.) illness as they believed it would be a beneficial variable to include when comparing the effectiveness of each palliative care arm (Preparatory Phase).

##### Research team action

Based on the recommendation to measure caregiver’s coping mechanisms, the research team incorporated the validated Zarit Caregiver Burden Interview into the initial assessment and subsequent patient follow-up surveys at 3, 6 and 12 months [10, 11].

##### SAC recommendation

The SAC suggested the EMPallA trial address loneliness, a specific concern of the seriously ill older adult population (Execution Phase).

##### Research team action

As a result, the research team incorporated the validated University of California Los Angeles (UCLA) Three-Item Loneliness Scale into the 3, 6 and 12-month patient follow up surveys to measure loneliness [8, 12, 13]. This added an additional quality of life measure to reflect intervention effects.

##### SAC recommendation

Due to project start up timeline and logistic barriers, the initial study design solely included English speaking patients. The SAC strongly recommended the EMPallA trial expand enrollment inclusion criteria to include Spanish-speaking patients as this would assist with increasing the generalizability and minimize disparities (Execution Phase).

##### Research team action

To effectively make this change the research team leveraged the original SAC members to specifically recruit a Latinx community partner to join the SAC. Once the Latinx SAC member was recruited, they assisted in developing Spanish patient-facing materials to be submitted to the IRB for approval. This recommendation also required the research team collaborate with each Spanish-speaking recruitment site to ensure a native Spanish-speaking RC was available for the enrollment and consent process to ensure cultural and linguistic sensitivity. The research team also updated their protocol to incorporate the use of a translator phone in recruitment and intervention delivery when a native Spanish-speaking team member was unavailable. This major study modification focused on ensuring the study population was representative of patients that come into the ED with life-limiting illnesses.

##### SAC recommendation

The SAC expressed an interest in ensuring data collection instruments were included to address patients’ and caregivers’ health as a whole. SAC members also continued to express the need to prioritize the patient perspective and requested the research team remain sensitive to conditions that impact the specific study participants, such as Alzheimer’s Disease and Dementia which may have been uncovered after enrollment (Execution Phase).

##### Research team action

As a result from these conversations, the validated, 10-item Patient-Reported Outcome Measurement Information System (PROMIS-10) was incorporated, with questions specific to caregiver physical, mental, and social health [14]. In this screening tool, a dementia question which indicates whether dementia was included in the patient’s active problem list was also documented. This question excluded participants at baseline, however, if a participant developed dementia throughout the course of the study, RCs were trained to use a dynamic approach to obtaining data from this population. For example, RCs prioritized certain questions within the follow-up surveys or requested the presence of a caregiver during survey completion to aid patients in their responses.

##### SAC recommendation

During the November 16, 2020, meeting, the SAC recommended the specialty, outpatient palliative care intervention arm expand services to be delivered via telehealth, due to the COVID-19 pandemic and subsequent epidemiological waves occurring throughout the country (Execution Phase).

##### Research team action

The research team collaborated with each of the subcontracted enrollment and implementation sites to understand state, local, and health system policies and procedures for delivering palliative care via telehealth and requested this service be offered for patient safety to EMPallA enrolled patients. From there, each local RC coordinated with the Outpatient Co-Investigator to understand and adapt the research protocols to local clinic scheduling practices. The expansion of providing a telehealth visit option to those enrolled in the specialty outpatient palliative care arm was a major protocol change for the EMPallA trial.

### Subtheme: recommendation for supplementary study materials

#### SAC recommendation

Prior to beginning recruitment, the SAC members reviewed a draft of the EMPallA Enrollment Welcome Packet and recommended changes to the language, length, and overall appearance (Execution Phase).

#### Research team action

The research team updated the Enrollment Welcome Packet to ensure it was written in plain language and incorporated the SAC member’s feedback regarding terminology and the use of specific palliative care words and phrases. Additionally, the SAC assisted in ensuring the materials were sensitive to the prospective study participants. They assisted in identifying which stock images would be most inclusive to use on the recruitment materials. Lastly, they assisted in partnering with the research team to ensure the consent process and benefits of palliative care were appropriately described in the developed of patient-facing materials.

#### SAC recommendation

Halfway through the EMPallA study, all sites were experiencing challenges in reaching targeted recruitment goals. To overcome this barrier, the SAC recommended the research team revisit and update previously developed patient-facing documents to emphasize what palliative care means and outline the goals and importance of the EMPallA study (Execution Phase).

#### Research team action

A one-page plain language, large font poster designed for older adults describing palliative care benefits and services was developed and incorporated into the recruitment packet materials.

### Theme 2—pragmatic

The Pragmatic theme was related to practical changes that could be made to deliver the intervention more effectively. All SAC recommendations within this theme were related to implementation advice such as suggestions and feedback to facilitate implementation strategies.

#### SAC recommendation

During the November 16, 2020, meeting, the research team expressed challenges in reaching caregiver recruitment goals and requested this meeting focus on potential strategies and solutions for overcoming set barriers. The SAC members encouraged the research team to focus on strategies related to increasing research coordinators (RCs) confidence and expertise on the topic area (e.g., implementing more role-playing trainings) and collaborating on enhancing patient/caregiver recruitment related materials (Execution Phase).

#### Research team action

RCs were re-trained to not only use more sensitive language, but also to emphasize the importance of EMPallA and the feasibility of caregiver responsibilities in the study. In addition, study materials were updated to be more inclusive of the challenges caregivers may experience when caring for someone with a life-limiting illness. Examples of using more compassionate and empathetic language posed by the SAC included using terms and phrases such as “supportive care,” “companion,” and “support system” instead of “caregiver” and “caregiver burden”.

#### SAC recommendation

The COVID-19 pandemic posed multiple challenges to patient recruitment and retention due to the lack of in-person contact between the research teams and patients. The research team heard feedback during telephonic follow-up survey calls that patients often did not remember the initial recruitment call. To aid in retention, the SAC recommended implementing re-introductory phone calls after initial recruitment to remind patients of the study goals, timelines, and expectations. (Execution Phase).

#### Research team action

To increase engagement and retention, the research team incorporated refresher calls (1 month after initial recruitment) to study participants to remind them of the study goals and expectations.

### Theme 3—Resource

The Resource theme was related to suggestions and recommendations that the SAC members provided specifically leveraging resources to enhance patient-centeredness.

#### SAC recommendation

After the trial had received approval for recruitment of Spanish-speaking patients, the SAC suggested the research team increase cultural competence by dedicating more training and learning opportunities for the RCs who were responsible for patient recruitment and enrollment (Execution Phase).

#### Research team action

RCs participated in meetings with a SAC member of Latinx descent to conduct mock recruitment/enrollment training sessions to ensure RC cultural and linguistical sensitivity. The RCs also participated in ongoing peer-to-peer learning collaboratives. This enhanced the RCs’ ability to effectively enroll by building trust and rapport with a diverse, seriously ill population.

### Theme 4—Dissemination

The final theme, Dissemination was specifically related to recommendations having to do with dissemination advice. The timeline of the study and partnership with the SAC likely impacted the number of recommendations emerging within this theme.

#### SAC recommendation

The SAC encouraged the research team to keep the enrolled patient and caregivers informed throughout the active study. They suggested the research team develop a public website for participants to see enrollment numbers, target goals, and study results. (Translation Phase).

#### Research team action

Unfortunately given the scope of the study the research team was unable to move forward with this suggestion. The research team did explore potential avenues for dissemination and communicated with their local IRB to see if it would be appropriate to house such a website, but it was deemed not appropriate until study completion. A mutual agreement was decided upon between the SAC group and the research team to disseminate plain language study results via mail in both English and Spanish to all participants after the study analysis had concluded. The SAC assisted in drafting this one-pager of study results and approved the final version that was disseminated to all participants.

## Discussion

### Patients, caregivers, and community as research partners, not simply participants, the SAC as a model for future trials

Although previous literature on best practices for engaging in participatory research has identified the need to involve stakeholders throughout the research process, there is limited literature illustrating the consistent impact of stakeholder involvement at each stage of the research process [15–18]. Literature on stakeholder involvement in clinical research has also been largely descriptive and lacking clear examples of how stakeholder involvement directly ties to changes in research procedures [19]. Our results demonstrate how continual involvement of a SAC in a clinical trial generated significant recommendations and IRB modifications from beginning to later stages of a large trial. Our SAC members assisted in providing key recommendations related to the scientific design, development of study materials, pragmatic implementation, prioritization of leveraging resources to enhance patient-centeredness, and dissemination.

EMPallA’s partnership with the SAC provides an example of successful patient and community incorporation in the conduct of a major multicenter randomized controlled trial. Multiple meetings over years resulted in many recommendations, over half of which resulted in IRB modifications. Most SAC recommendations in the first meeting were related to the execution phase of the trial, specifically in the study recruitment stage. As the trial progressed, the largest proportion of the SAC recommendations were still associated with the execution phase but shifted toward data collection stage within this phase. The consistent engagement of the SAC throughout the entire research process allowed real-time problem solving and led to meaningful changes at all phases of the trial. The SAC made the most impact in the execution phase of the project, specifically in the stages of study design and study recruitment. Many of the execution phase recommendations were related to both the science and pragmatic themes. The SAC improved study recruitment and retention by improving inclusivity and representation and by focusing on the patient perspective [15]. Overall their recommendations addressed accessibility of trial materials, cultural competency of research personnel, diversity of our participants, design of patient-facing materials, measurability of study variables, and study design and protocol. When possible, the research team made every effort to incorporate all the SAC recommendations and advice. However, It is important to note not all SAC’s suggestions led to formal IRB modifications in the study due to feasibility, study restrictions, and the privacy of the participants. Additionally, some recommendations could be made but did not require a formal IRB modification. Typically, formal IRB modifications are only required if items such as inclusion/exclusion criteria, procedures, recruitment, consent forms, questionnaires are modified. The research team always consulted their local IRB if they had any questions related to if they needed to submit a formal modification or not.

Through their focus on inclusivity and cultural competency, the SAC made suggestions improving study recruitment, a major tangible benefit to the study [20]. In particular, older adults, especially those with substantial health problems, can be difficult to recruit to research and continue to be underrepresented in clinical research despite their increasing population across all demographics in the United States [21, 22]. Key approaches identified in recruiting and retaining patients include early in-depth planning, study advisory boards, and a sensitive approach to eligible patients [22]. This may motivate funders and systemic support for SAC involvement in research [23]. Bringing the patient perspective into recruitment led to practical suggestions for creating clearer, plain language patient facing materials and processes.

Our experience demonstrates how engaging a SAC can be a successful method for improving inclusivity, accessibility, and representation in clinical trials [24]. Improving cultural competence and inclusion in clinical trials is crucial for translating clinical research into the real-world practice. A 2021 review of clinical trial literature identified three main recommendations for promoting inclusion in clinical trials: improving the cultural competency and sensitivity of all clinical trial staff, establishing a diverse community advisory panel, and increasing recruitment of staff from under-served groups [20]. The EMPallA SAC accomplished these recommendations by advocating for inclusion and recruitment of caregivers in research [20], and by increasing access to translated documents and translation services. Integrating these strategies benefited the overall study. Members serving on the SAC were of different ethnic groups and backgrounds, providing diverse inputs from various cultures and religions.

Shippee and colleagues [9] also delineate four components of patient and public involvement in research that are beyond the levels of patient and service user engagement in research. These four components are: 1. patient and service user initiation, 2. building reciprocal relationships, 3. co-learning, and 4. re-assessment and feedback. Components 2–4 were the focus of the SAC’s research involvement since their project involvement occurred after the grant was obtained. While the research team’s activities related to these four components are outside the scope of this article and will be described elsewhere, the research team supported the SAC members’ diversity by ensuring inclusive equitable access to the research process. Examples include ensuring study materials were in large-font printed materials, close captioning during videoconferencing calls, and travel arrangements for reduced mobility when meeting in person [5].

### Limitations

We measured the impact of the SAC using study adjustments and modifications as a metric but were unable to quantitatively evaluate how the recommendations impacted patient recruitment, retention, or study outcomes. For example, once we implemented a change, we did not have a control group to test the effectiveness (i.e. we could not quantify if updating our study materials increased recruitment or not). Future multi-method research should be performed to explore how stakeholder engagement influences the experience of participants in similar trials. This could be accomplished via surveys, focus groups or other research methodologies to participants regarding stakeholder-recommended changes and their overall study experiences.

We did not identify which recommendations came from specific people or stakeholder groups. Based on our lived experiences, conversations during SAC meetings were often led by the patient and caregiver members. While their voice and recommendations were extremely valuable in improving the patient’s experience in research, it is possible other groups were not able to contribute to the same degree. To ensure all stakeholder groups’ feedback was considered we circulated the notes immediately after the meeting and provided all participants an opportunity to individually write via e-mail any questions/comments/concerns/reflections. Other SACs have structured their meetings by separate groups which is more labor intensive but could lead to focused involvement of all stakeholder groups [24]. We intentionally chose to not silo the groups as we felt there would be incredible value (brainstorming ideas, learning from each other, networking etc.) in meeting consistently as one large group and creating a sense of trust and community. Reflecting back, at times a mixed structure approach where meetings are held partially with the entire SAC, and in three individual groups, could have been beneficial to accomplish the desired outcomes. Future research should explore the ideal format of SAC meetings with individuals from different backgrounds.

Few recommendations were made in the preparatory and translation themes of the Shippee, et al., framework. First, the lack of recommendations pertaining to the preparatory phase can be attributed to the nature of a peer-reviewed grant. Specifically, the Principal Investigator (CRG) was primarily responsible for writing the grant and setting the research agenda. However, CRG did collaborate with several of the SAC members before the official formation of the committee to gain insight when writing the initial application. It would have been helpful to have had SAC input earlier in the preparatory phase, but there was no mechanism to reimburse them for their time prior to obtaining the grant funding. However, future studies should explore potentially a volunteer SAC earlier in the grant writing phase. Moreover, at the time of this thematic analysis the EMPallA trial was currently in the data analysis phase of the overall parent project, therefore fewer conversations about the translational phase stages had been discussed. However, we anticipate more translational phase recommendations by the SAC will emerge as the implementation phase ends.

## Future Implications

﻿As more studies incorporate “patient-centered” foci stakeholders may offer more pragmatic ways to ensure multiple, diverse patient perspectives are integrated into research [1]. Future research should utilize the patient perspective in a direct manner and quantify its effectiveness in contributing to the value of the trial. Further analysis of the impact of patient and community stakeholders on the translational phase of the trial could provide a complete picture of the efficacy of the SAC in this analysis. This will give insight into how stakeholders can impact the adoption of study findings into standard care. In addition, the perspective of the SAC members on their efficacy should be evaluated, as well as that of the research team's opinions of the SAC to evaluate cooperation and satisfaction between these two groups.

## Conclusion

Actively engaging patient and community stakeholders in clinical trials offers a strategic way to ensure the conduct of a clinical trial is patient-centered, addresses unmet clinical needs, and facilitates patient recruitment and retention. The SAC has demonstrated to be not only feasible, but instrumental in the design and conduct of EMPallA. Overall, the SAC possessed the ability to positively advocate for patient safety, cultural competence, representation, and protection of vulnerable populations.

## Data Availability

All data generated or analyzed during this study are included in this published article.

## Abbreviations

PCORI: Patient-Centered Outcomes Research Institute
SAC: Study Advisory Committee
ED: Emergency Department
EMPallA: Emergency Medicine Palliative Care Access
IRB: Institutional Review Board
NYU: New York University
PI: Principal Investigator
SRPM: Senior Research Project Manager
RC: Research Coordinator
RCT: Randomized Controlled Trial
PROMIS-10: Patient-Reported Outcome Measurement Information System
UCLA: University of California Los Angeles
US: United States
